# Volume-based algorithm of lung dose optimization in novel dynamic arc radiotherapy for esophageal cancer

**DOI:** 10.1038/s41598-021-83682-3

**Published:** 2021-02-23

**Authors:** Kuan-Heng Lin, Chen-Xiong Hsu, Shan-Ying Wang, Greta S. P. Mok, Chiu-Han Chang, Hui-Ju Tien, Pei-Wei Shueng, Tung-Hsin Wu

**Affiliations:** 1 Department of Biomedical Imaging and Radiological Sciences, National Yang Ming Chiao Tung University, Taipei, Taiwan; 2 Division of Radiation Oncology, Far Eastern Memorial Hospital, New Taipei City, Taiwan; 3 Industrial Ph.D. Program of Biomedical Science and Engineering, National Yang Ming Chiao Tung University, Taipei, Taiwan; 4 Department of Nuclear Medicine, Far Eastern Memorial Hospital, New Taipei City, Taiwan; 5 Biomedical Imaging Laboratory, Department of Electrical and Computer Engineering, Faculty of Science and Technology, University of Macau, Macau, SAR China; 6 Faculty of Medicine, School of Medicine, National Yang Ming Chiao Tung University, Taipei, Taiwan

**Keywords:** Oesophageal cancer, Radiotherapy

## Abstract

This study aims to develop a volume-based algorithm (VBA) that can rapidly optimize rotating gantry arc angles and predict the lung V_5_ preceding the treatment planning. This phantom study was performed in the dynamic arc therapy planning systems for an esophageal cancer model. The angle of rotation of the gantry around the isocenter as defined as arc angle (θ_A_), ranging from 360° to 80° with an interval of 20°, resulting in 15 different θ_A_ of treatment plans. The corresponding predicted lung V_5_ was calculated by the VBA, the mean lung dose, lung V_5_, lung V_20_, mean heart dose, heart V_30_, the spinal cord maximum dose and conformity index were assessed from dose–volume histogram in the treatment plan. Correlations between the predicted lung V_5_ and the dosimetric indices were evaluated using Pearson’s correlation coefficient. The results showed that the predicted lung V_5_ and the lung V_5_ in the treatment plan were positively correlated (r = 0.996*, p* < 0.001). As the θ_A_ decreased, lung V_5_, lung V_20_, and the mean lung dose decreased while the mean heart dose, V_30_ and the spinal cord maximum dose increased. The V_20_ and the mean lung dose also showed high correlations with the predicted lung V_5_ (*r* = 0.974, 0.999, *p* < 0.001). This study successfully developed an efficient VBA to rapidly calculate the θ_A_ to predict the lung V_5_ and reduce the lung dose, with potentials to improve the current clinical practice of dynamic arc radiotherapy.

## Introduction

Acute radiation pneumonitis is one of the major morbidities after radiotherapy for esophageal tumors^1–4^. Dynamic arc radiotherapy is currently the most common radiotherapy technique, which involves rotation of the gantry of a linear accelerator for 360° around the isocenter of the tumor to administer intensity-modulated radiation and achieve high tumor conformity^5,6^. However, the higher the conformity is, the bigger the angle of the radiation beam required, consequently causing radiations spread to organs at risk such as the lungs, heart and spinal cord^7,8^. Therefore, the selection of gantry arc angle and dose constraints are crucial during the radiation treatment planning (RTP). The treatment plan should prescribe sufficient dose to achieve the therapeutic effect and fulfil the dose constraints of organs at risk^9^.

The selection of gantry arc angle and dose constraints might differ based on the clinical experience and trial-and-error approaches from radiation oncologists and medical physicists for dynamic arc radiotherapy in the current computerized treatment planning systems. Therefore, a crucial consideration in dynamic arc radiotherapy is to determine the optimal arc angle while optimizing the RTP. The idea of the fan-shaped complete block (FSCB) was first proposed by Chang et al.^10^, which was designed to limit the beam angle and reduce lung dose in helical tomotherapy (HT). However, studies on the angle of the FSCB have only been explored at HT rather than the novel dynamic arc radiotherapy. Moreover, no applicable methods have been developed to rapidly optimize the arc angle of the gantry, meaning that radiation oncologists and medical physicists must manually determine arc angles for each RTP based on their experiences. Repeated computation, testing and lung dose analysis required for obtaining optimal angles are time-consuming and prone to human errors. Thus, this study aims to develop a novel volume-based algorithm (VBA) that can rapidly optimize the arc angles of rotating gantry and predict the relative lung volume receiving more than 5 Gy (V_5_) preceding the inverse planning in the dynamic arc radiotherapy planning systems.

## Materials and methods

### Phantom image acquisition and delineation of planning target volume and organs at risk

**Figure 1:**
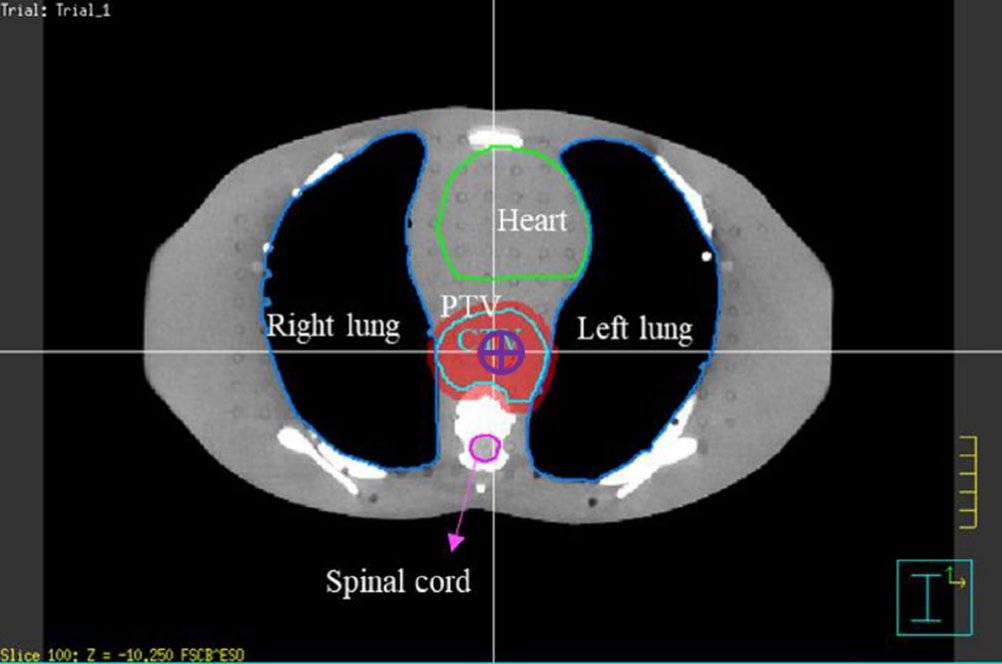
The axial view of the virtual esophageal tumor in the anthropomorphic phantom. The green line region represents the heart, the pink line region represents the spinal cord, and the dark blue line regions represent the lungs. The light blue line region represents the CTV and the red area represents the PTV.

An anthropomorphic phantom study was simulated in the dynamic arc therapy planning systems for an esophageal cancer model. An anthropomorphic phantom (ATOM 701; CIRS, Norfolk, VA, USA) was scanned using a computed tomography (CT) (Discovery CT590 RT, GE Medical Systems, Amersham, UK). The slice thickness of CT image was 2.5 mm, and the scan range was from the oral cavity to the L5 vertebra. The CT images were then imported to the Pinnacle treatment planning system (version 9.8; Philips Medical Systems North America, Andover, MA, USA) to delineate the virtual esophageal tumor and surrounding normal organs in each slice. The location of the virtual esophageal tumor was set in the thoracic middle-third esophagus; the horizontal diameter and vertical axis length of the virtual gross tumor volume (GTV) were 4.4 cm and 11.4 cm respectively. The clinical target volume (CTV) was designed to cover a region with subclinical disease from GTV by expanding 4 cm superiorly and inferiorly, and 0.5 cm left, right, anteriorly and posteriorly. To define the planning target volume (PTV), organ movements caused by breathing, swallowing and position uncertainty in each therapy were considered. In accordance with clinical experience, the PTV was defined by expending the CTV three-dimensionally by 0.8 cm to the superior, inferior, left, right, anterior and posterior. The horizontal diameter, vertical axis length and total volume of the PTV were 7 cm, 21 cm and 497.73 cm^3^, respectively. The normal organs such as heart, lung and spinal cord were defined (Fig. 1).

### Definition of the arc angle and the restricted angle of VBA

**Figure 2:**
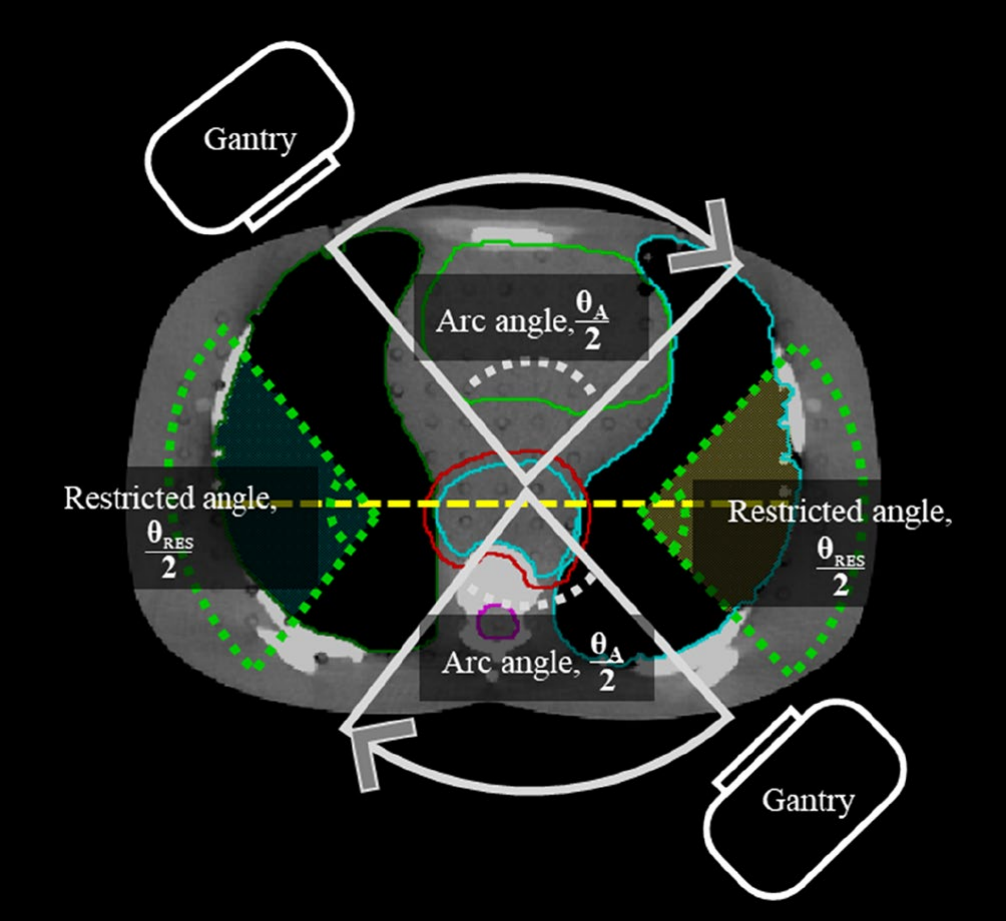
The gantry’s arc angle θ_A_ (grey solid line) and the θ_RES_ (green dotted line) defined in dynamic arc therapy.

This study used the volumetric modulated arc therapy (VMAT) and the HT system to simulate treatment for esophageal cancer. The centroid of the PTV was defined as the isocenter. The angle of rotation of the gantry around the isocenter was defined as arc angle (θ_A_) and the remaining angle was the angle of restricted radiation, defined as the restricted angle (θ_RES_) (Fig. 2 and Eqs. 1–2).

1 $$\uptheta _{{{\rm A}}} + \,\uptheta _{{{{\rm RES}}}} = 360^{\circ}$$

The relationship between restricted angle in left or right lung, θ_RESL_ or θ_RESR_ and θ_RES_ was shown below.

2 $$\uptheta _{{{\text{RESL}}}} + \,\uptheta _{{{\text{RESR}}}} = \,\uptheta _{{{\text{RES}}}}$$

### The establishment volume-based algorithm (VBA) for treatment planning

**Figure 3:**
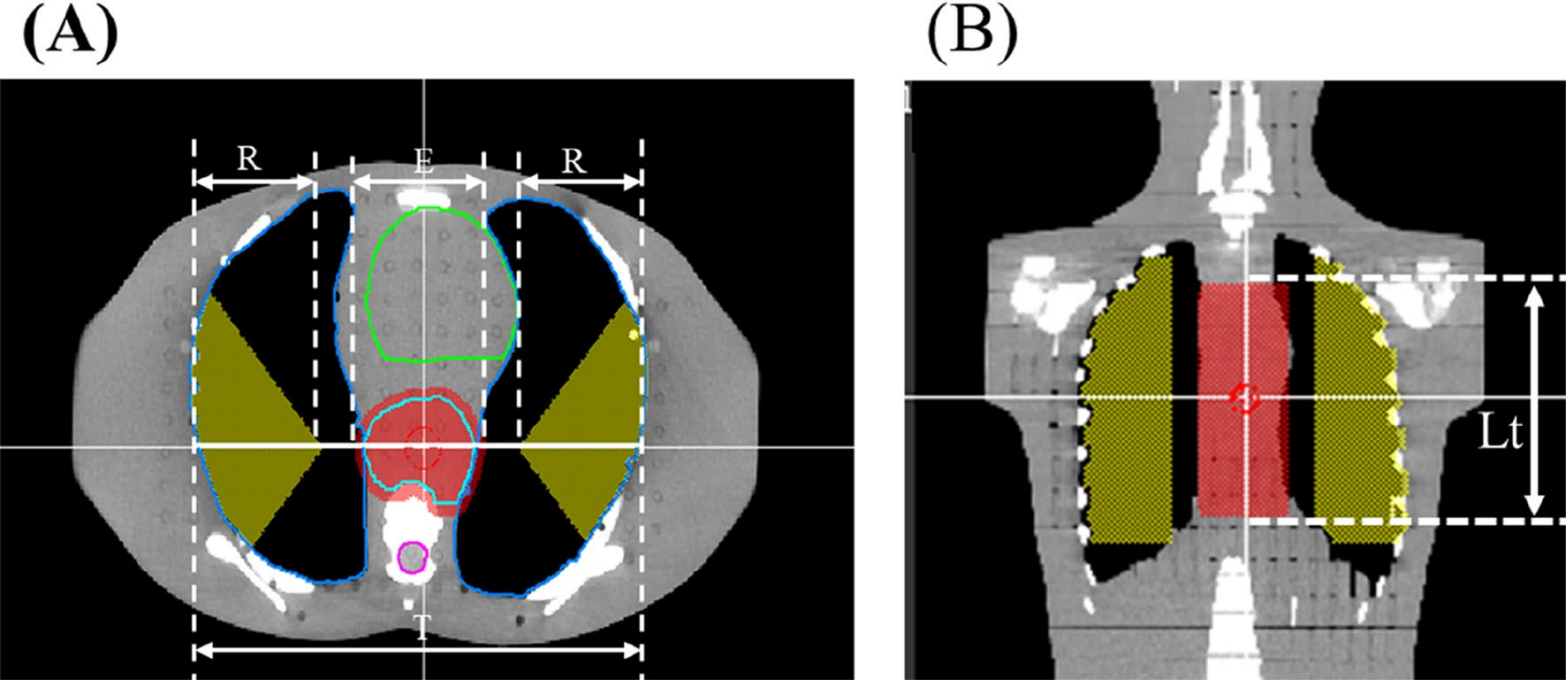
(**A**) Axial view and (**B**) coronal view of the PTV (red area) and restricted volume (yellow area). The transverse diameter of the thorax (T), the radius of one side of the restricted volume (R), the transverse diameter of the PTV (E) and the length of the PTV (Lt) are defined in the images.

As illustrated in Fig. 3, the transverse diameter of the thorax (T) and the diameter of the PTV (E) were measured on the axial plane of the centroid of the PTV (Fig. 3A), while the vertical axis length of the PTV (Lt) was measured on the coronal image of the centroid of the PTV (Fig. 3B).

The radius of one side of the restricted volume (R) was calculated by Eq. (3):

3 $${\text{R }} =\frac{{{\text{T}}-{\text{E}}-4}}{2}$$

**Figure 4:**
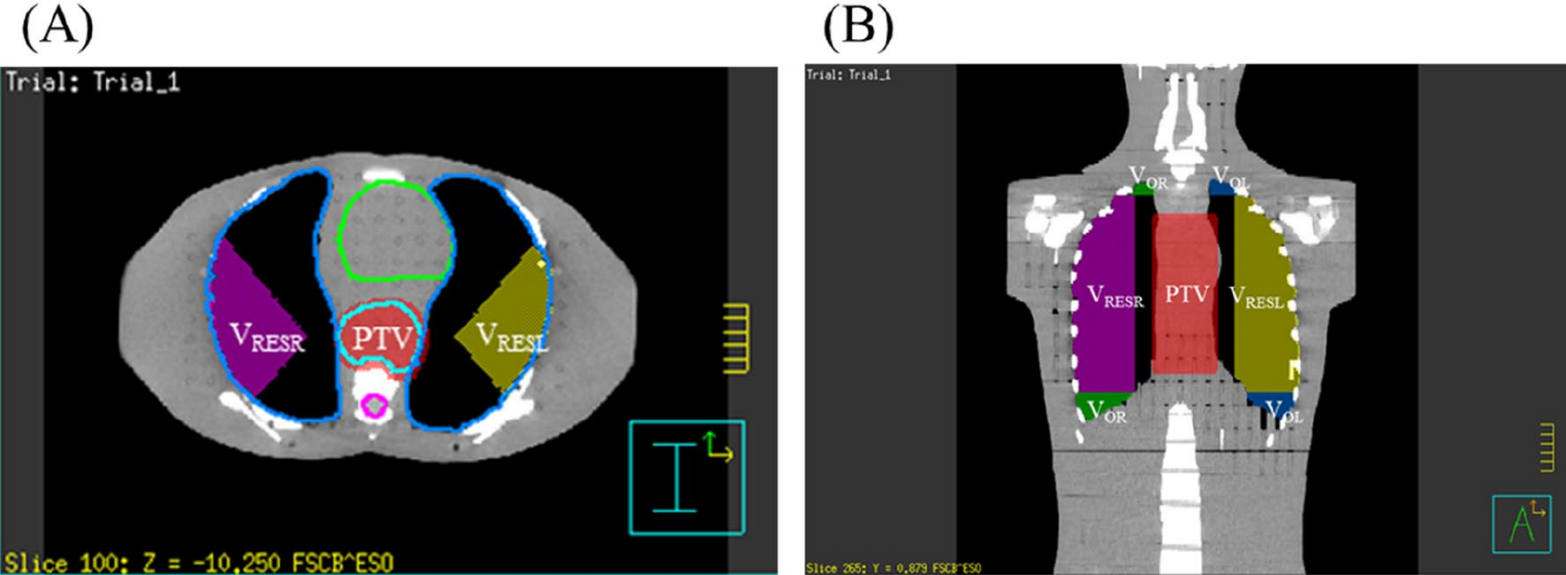
(**A**) Axial view and (**B**) coronal view of different volumes of interest using volume-based algorithm (VBA). Restricted volume (V_RES_) was divided into right lung restricted volume (V_RESR_) (purple area) and left lung restricted volume (V_RESL_) (yellow area). The total volume out of the field (V_OW_) was the sum of the volume out of the field in the right lung (V_OR_) (green area) and the volume out of the field in the left lung (V_OL_) (blue area). The non-radiated volume (V_NR_) was the sum of V_RES_ and V_OW_. The rest of the lung volume was defined as the radiated lung volume.

The θ_RES_ were determined for each slice of image according to the defined θ_A_. Eventually, a fan volume was simulated. The volume which the fan volume overlapped with the lung was defined as the restricted volume (V_RES_) (Fig. 4). The total volume out of the field (V_OW_) was the sum of the volume out of the field in the right lung (V_OR_) and the volume out of the field in the left lung (V_OL_). The combination of V_RES_ and V_OW_ was defined as the non-radiated volume (V_NR_) in the lungs (Eq. 4) and the rest of the lung volume was defined as the radiated lung volume. The whole lung volume was defined as V_W_.

4 $${\text{V}}_{{{\text{NR}}}} = {\text{ V}}_{{{\text{RES}}}} + {\text{V}}_{{{\text{OW}}}}$$

The R, Lt and θ_RES_ are then input into Eq. (5) to obtain the fan volume of V_RES_:

5 $${\mathrm{V}}_{\text{RES}}=\uppi {\mathrm{R}}^{2}\frac{{\uptheta }_{\mathrm{RES}}}{{360}^\circ }(\text{Lt}+4)$$

**Figure 5:**
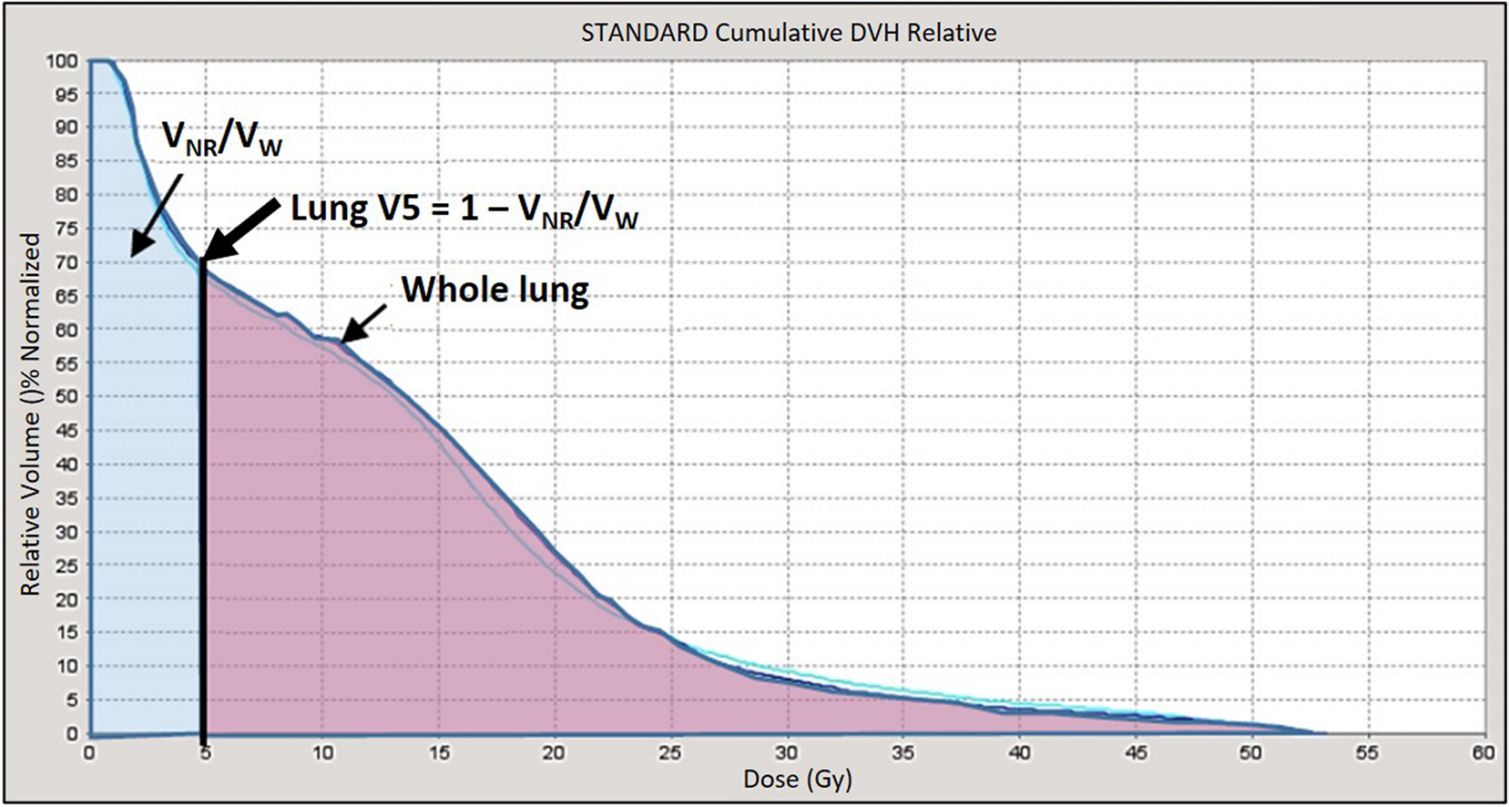
The dose-volume histogram for lung.

As presented in the dose–volume histogram (DVH) (Fig. 5), the area of radiation dose < 5 Gy represented the proportion of V_NR_ to the whole lung in the treatment plan, V_NR_/V_W_. On the contrary, the lung V_5_ is the proportion of the radiated lung volume with radiation dose ≥ 5 Gy to the whole lung in the treatment plan, 1 − V_NR_/V_W_.

On the basis of the lung dose constraint study by Pinnix et al.^11^, the anticipated starting point of lung V_5_ in this study was set to 55%; that is, more than 45% of the V_W_ was defined as the nonradiated volume (V_NR_, Eq. 6).

6 $${\text{V}}_{{{\text{NR}}}} \ge {\text{V}}_{{\text{w}}} \times \, 0.{45}$$

Equations (4) and (5) are input into Eq. (6) to produce Eq. (7):

7 $$\uppi {\mathrm{R}}^{2}\frac{{\uptheta }_{\mathrm{RES}}}{360^\circ }(\mathrm{Lt}+4)+{\mathrm{V}}_{\mathrm{OW}}\ge {\mathrm{V}}_{\mathrm{W}}\times 0.45$$

**Figure 6:**
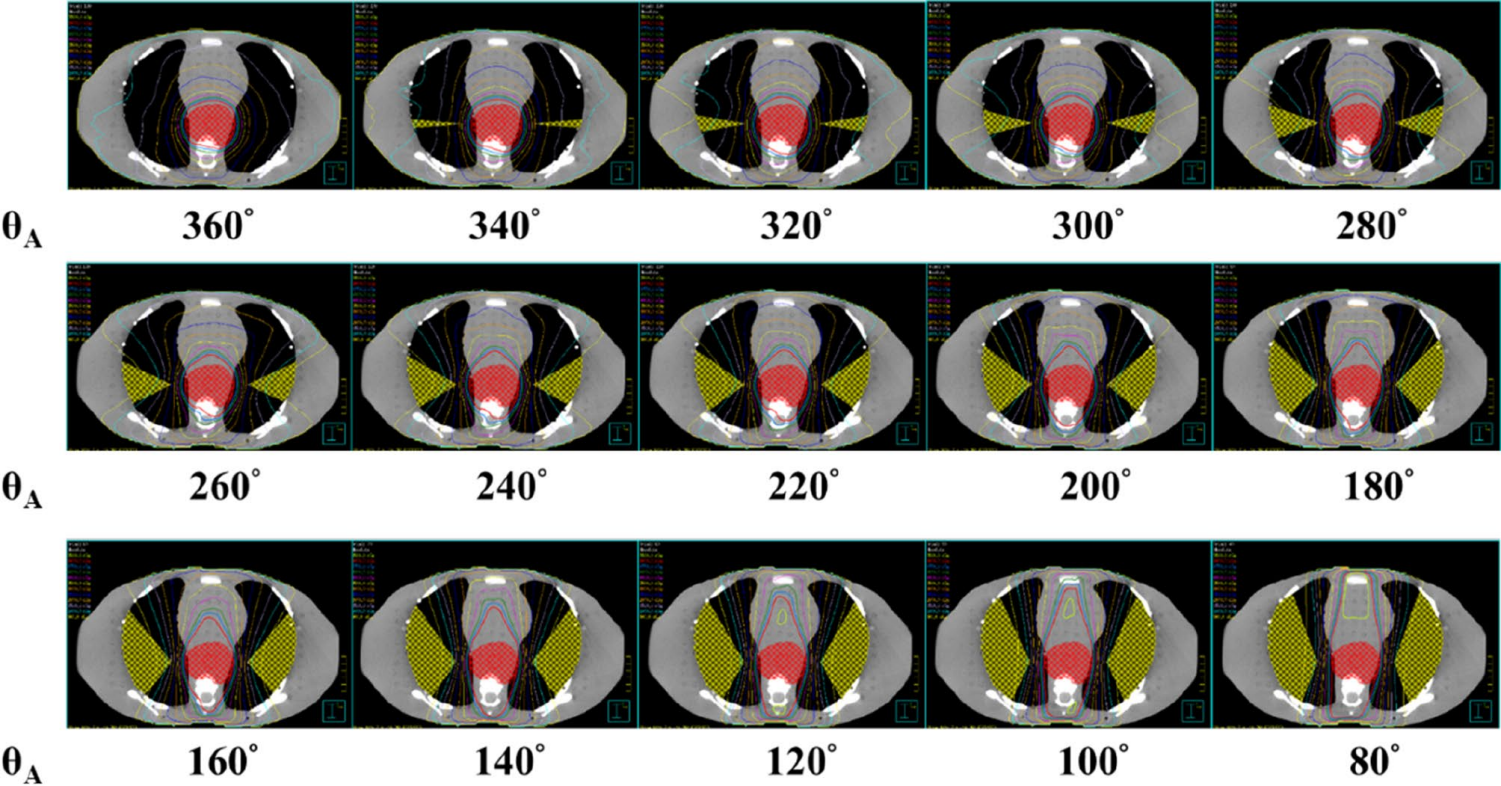
Fifteen arc angles (θ_A_) range from 360° to 80° with 20° interval in RTP. The corresponding V_RES_ (yellow area) are established in the both lungs.

The θ_A_ ranged from 360° to 80° with an interval of 20°, resulting in 15 RTP (Fig. 6). Corresponding θ_RES_ of 0° to 280° and V_RES_ was established in the two lungs. The equations of the VBA were used to calculate V_RES_, V_NR_ and the predicted lung V_5_. During the VBA calculation, transverse diameter of the thorax (T), the transverse diameter of the PTV (E) and the length of the PTV (Lt) were set to be 30 cm, 7 cm and 21 cm, respectively. Moreover, the V_W_ and V_OW_ were set to be 4483.38 and 294.72 cm^3^, respectively for this particular phantom. The θ_A_ would be set in VMAT and the angle of complete block would be set with θ_RES_ in HT. Herein, 100% of the prescribed dose was received by 100% of the CTV while 95% of the prescribed dose was received by 95% of CTV. Then, RTP of 15 different θ_A_ were performed in HT and VMAT separately with 20 iterations and 40 iterations. A total of 30 HT and 30 VMAT RTP were generated. Finally, the mean lung dose, lung V_5_, lung V_20_, mean heart dose, heart V_30_, the spinal cord maximum dose and conformity index (CI) were assessed in DVH. The CI was calculated by the definition of Radiation Therapy Oncology Group^12^.

### Statistical analyses

The following parameters were recorded using the information provided by the cumulative DVH in the RTP of HT and VMAT: mean lung dose, lung V_5_, lung V_20_, mean heart dose, heart V_30_, the spinal cord maximum dose and CI. SPSS software package version 24.0 (IBM Corporation., Armonk, NY, USA) was used to conduct a Pearson correlation analysis between the predicted lung V_5_ by VBA and the radiation dose of various normal tissues in the treatment plan. A *p* < 0.01 was considered as statistically significant.

## Results

### Relationship between the predicted lung V_5_ by VBA and the lung V_5_ in the treatment plans

Table 1 shows for 15 different θ_A_, corresponding θ_RES_, V_RES_, V_NR_, the predicted lung V_5_ by VBA (V_5__VBA) and the lung V_5_ in the treatment plan (V_5__RTP). When θ_A_ was 360°, the θ_RES_, V_RES_, V_NR_/V_W_, V_5__VBA and the lung V_5__RTP were 0°, 0 cm^3^, 6.75%, 93.43% and 92.37%, respectively. When θ_A_ was 80°, the corresponding θ_RES_, V_RES_ and V_NR_/V_W_ were 280°, 2230 cm^3^, 56.32% while the corresponding lung V_5__VBA decreased to 43.68% and the lung V_5__RTP decreased to 44.48%. When the θ_A_ was no more than 120°, either the lung V_5__VBA or the lung V_5__RTP would be less than 55%. Moreover, the differences between the lung V_5__VBA and the lung V_5__RTP over all θ_A_ were less than 5%.

**Table 1:** The 15 different θ_A_, the lung V_5__VBA and the lung V_5__RTP.

θ_A_ (°)	θ_RES_ (°)	V_RES_ (cm^3^)	V_NR_/V_W_ (%)	Lung V_5__VBA (%)	Lung V_5__RTP (%)	Difference of Lung V_5__VBA and V_5__RTP (%)
360	0	0	6.57	93.43	92.37	− 1.14
340	20	128	9.43	90.57	90.65	0.09
320	40	249	12.12	87.88	89.43	1.76
300	60	377	14.99	85.01	87.92	3.41
280	80	508	17.90	82.10	85.46	4.08
260	100	642	20.89	79.11	82.31	4.05
240	120	789	24.17	75.83	78.09	2.99
220	140	946	27.67	72.33	74.46	2.95
200	160	1107	31.28	68.72	70.58	2.70
180	180	1274	34.98	65.02	65.90	1.36
160	200	1458	39.10	60.90	61.68	1.27
140	220	1634	43.02	56.98	56.36	− 1.09
120	240	1825	47.27	52.73	51.67	− 2.01
100	260	2013	51.47	48.53	47.79	− 1.53
80	280	2230	56.32	43.68	44.48	1.83

### Assessment of doses delivered to organs at risk and the conformity of plans at various θ_A_ in the treatment plans

There were 30 HT and 30 VMAT treatment plans calculated from 15 different θ_A_ as shown in Table 2. When θ_A_ was 360º, the mean lung dose, lung V_5_, and V_20_ were 18.40 Gy, 92.37%, and 32.21%, respectively, the mean heart dose and heart V_30_ were 18.59 Gy and 6.28%, respectively, and the spinal cord maximum dose was 50.87 Gy. When θ_A_ was reduced to 80°, the mean lung dose, lung V_5_, and V_20_ were 10.38 Gy, 44.48%, and 18.88%, respectively, the mean heart dose and heart V_30_ were 37.76 Gy and 72.77%, respectively, and the spinal cord maximum dose was 54.80 Gy. As θ_A_ decreased, the mean lung dose, lung V_5_, and lung V_20_ decreased, the mean heart dose, heart V_30_ and CI increased, while the spinal cord maximum dose slightly increased.

**Table 2:** Comparing 15 different θ_A_, normal tissue doses and conformity indices in the radiation treatment plans.

θ_A_ (°)	θ_RES_ (°)	V_RES_ (cm^3^)	Mean lung dose (Gy)	Lung V_20_(%)	Lung V_5_ (%)	Mean heart dose (Gy)	Heart V_30_ (%)	Spinal cord maximum dose (Gy)	CI Of HT	CI of VMAT
360	0	0	18.40	32.21	92.37	18.59	6.28	50.87	1.15	1.21
340	20	128	17.76	30.61	90.65	20.72	10.93	50.86	1.17	1.46
320	40	249	17.48	30.32	89.43	22.63	16.76	51.53	1.21	1.52
300	60	377	17.14	30.30	87.92	22.88	18.12	51.65	1.23	1.48
280	80	508	16.73	29.86	85.46	24.34	23.36	51.93	1.22	1.56
260	100	642	16.30	29.88	82.31	24.28	23.05	52.71	1.22	1.55
240	120	789	15.69	29.62	78.09	26.53	32.25	52.78	1.18	1.71
220	140	946	15.08	28.37	74.46	27.07	34.84	53.26	1.18	1.75
200	160	1107	14.47	27.45	70.58	28.43	42.80	53.42	1.19	1.99
180	180	1274	13.88	26.77	65.90	30.07	58.54	54.14	1.24	2.04
160	200	1458	13.16	25.14	61.68	31.86	60.33	55.52	1.21	2.26
140	220	1634	12.53	24.21	56.36	33.11	61.83	54.70	1.29	2.59
120	240	1825	11.61	22.15	51.67	34.71	65.50	54.70	1.33	2.90
100	260	2013	11.07	21.04	47.79	35.57	70.26	54.48	1.31	3.11
80	280	2230	10.38	18.88	44.48	37.76	72.77	54.80	1.34	3.58

**Figure 7:**
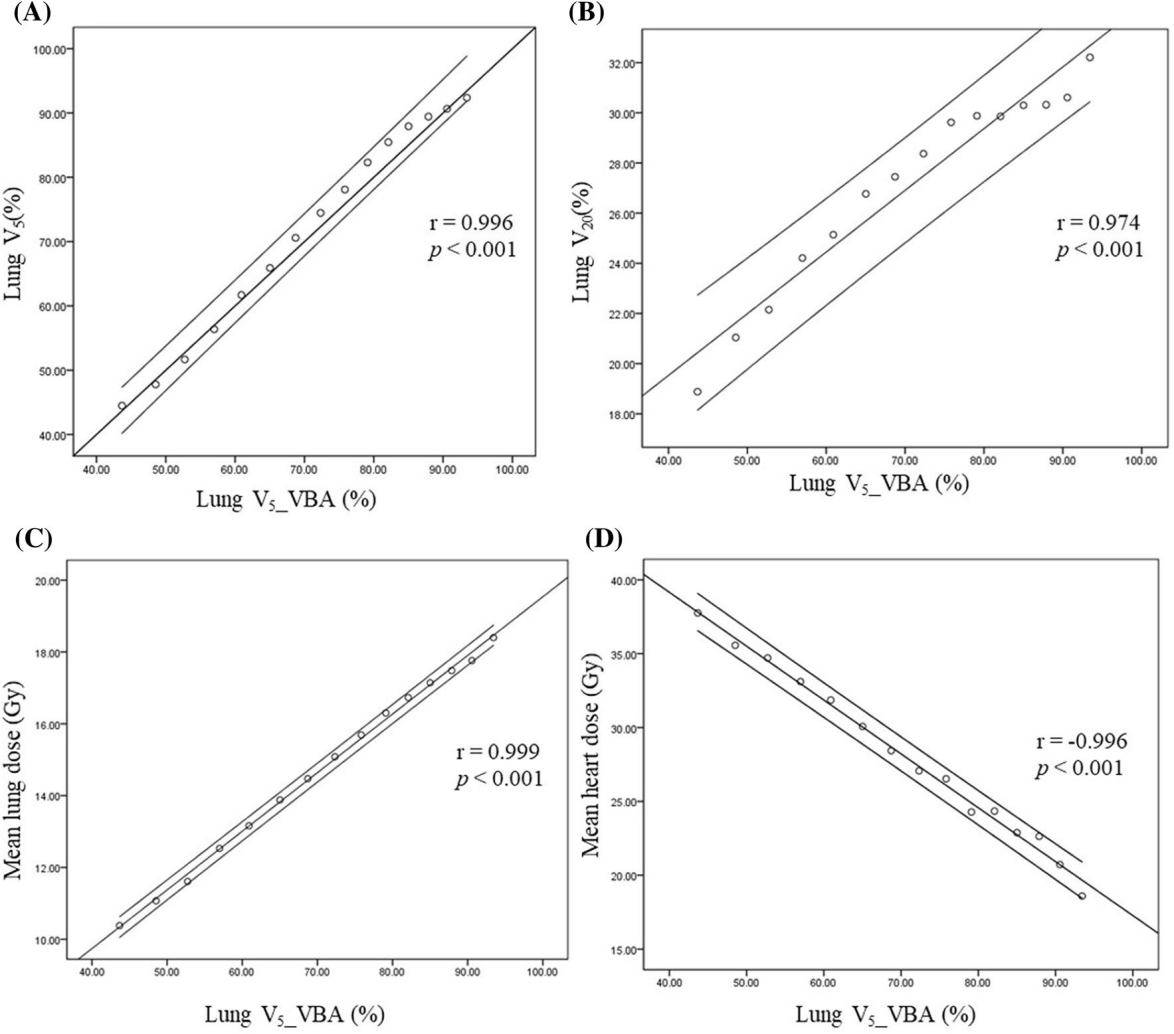
Pearson correlation coefficient between the lung V_5__VBA and the (**A**) lung V_5_; (**B**) lung V_20_; (**C**) mean lung dose; (**D**) mean heart dose in the treatment plans.

Figure 7 shows the correlation between the lung V_5__VBA at different θ_A_ and various normal tissue doses in the treatment plan. The lung V_5_ and V_20_ as well as the mean lung dose were significantly and positively associated (*r* = 0.996, 0.974, 0.999, *p* < 0.001) with the lung V_5__VBA (Fig. 7A–C). The mean heart dose was significantly and negatively correlated (*r* =  − 0.996, *p* < 0.001) with the lung V_5__VBA (Fig. 7D).

## Discussion

To our knowledge, the novel VBA was the first algorithm that developed to rapidly calculate the optimal gantry arc angle and precisely predict the proportion of the lung V_5_, especially preceding the RTP process for dynamic arc radiotherapy. Also, the lung V_5__VBA highly correlated with the V_5__RTP, demonstrating the effectiveness of the VBA to predict the lung V_5_ at 15 different θ_A_ from 80° to 360°.

Yin et al.^5^ demonstrated that when the mean lung V_5_ was higher than 80%, lung radiotoxicity might increase. Moreover, Wang et al.^13^ demonstrated that more lung volume can be protected by preventing it from receiving radiation doses of more than 5 Gy. The mean lung dose and V_5_ were highly related to the risk of radiation pneumonitis, i.e., 3% and 38% within 1 year for V_5_ < 42% and V_5_ > 42% respectively. In summary, the incidence of radiation pneumonitis was positively correlated with the mean lung dose, V_20_, V_10_, and V_5_. It is important to reduce the low dose distribution volume, to reduce the risk of complications. Song et al.^14^ analysed the correlation between lung dose and the level of lung inflammation in patients with lung cancer receiving HT. They suggested that the V_5_ in the other lung should be maintained at < 60% to reduce the risk of radiation pneumonitis. Pinnix et al.^11^ noted that a lung V_5_ exceeding 55% was associated with the maximum likelihood ratio for radiation pneumonitis. Thus, lung V_5_ was a crucial predictor of radiation pneumonitis. The algorithm developed in this study can be used to efficiently calculate the gantry arc angle to determine the optimal lung V_5_.

The advancement of radiotherapy treatment plans not only provided personalised management for each patient but also increased patient survival rates. However, treatment plan development was time-consuming and labour-intensive, since radiation oncologists and medical physicists must devise treatment plans with great caution to reduce damage to vital nerves, tissues and organs on the patients. Lin et al.^15^ indicated that it took an average of 3.8 h to manually complete a treatment plan with a full arc. However, many companies have developed various automatic treatment planning systems, such as the Pinnacle Auto-Planning and RapidPlan Knowledge-Based Planning software with the use of machine learning methods. Hansen et al.^16^ suggested that the average time required for the automated treatment planning system was 135 min plus about 20 min for the manual operation, i.e., a total of 155 min. More recently, Krayenbuehl et al.^17^ compared five automatic treatment planning systems, four of which completed RTP within 20 min. The calculation-intensive part of the automatic treatment planning system was the optimizing process. The PTV and all the normal tissues must first be selected, and the arc angle must be set before using the automatic treatment plan system to generated RTP of VMAT. Nevertheless, with our proposed algorithm, as soon as the length of the PTV was defined, the optimal arc angle corresponding to the expected lung V_5_ < 55% could be rapidly calculated within 5 min in the optimizing process of VMAT and HT.

Lauche et al.^18^ stated that both VMAT and HT provided treatment plans with high tumor comformality and could maintain dose deliverd to normal organ within constraints. Nevertheless, the algorithm developed in this study could be applied to both VMAT and HT to predict the lung V_5_ and calculate the corresponding gantry arc angles. In the VMAT treatment planning system, the optimal gantry arc angle would be defined before optimisation. If the VBA was applied to a HT treatment planning system, a complete block would be set in the lungs, and the θ_RES_ would be set to 360° − θ_A_ to control the radiation angle. When applied to the calculation of both VMAT and HT treatment planning system, the VBA effectively controled the lung V_5_.

Our study had some limitations. In clinical applications of VBA, variations such as the larger tumor length and extensive lymph nodes should also be considered. When the radiated field was too large to reach the expected lung V_5_, operators could follow as low as reasonably achievable (ALARA) principle and limit the radiation dose manually in the treatment plan. Furthermore, our phantom study simulated different θ_A_ in RTP (Table 2). In our study, the differences between the lung V_5__VBA and lung V_5__RTP were from 0.09 to 4.08%, which needed to be considered. However, the desired lung V_5_ could be achieved by dose constraints during the optimization. The doses of spinal cord were relatively higher than clinical practices which the constraint should be manually limited to < 45 Gy. The dose to heart increased as restricted angle increased. The doses of heart were also relatively higher than clinical practices in θ_A_ from 80° to 220°. Therefore, the dose to heart would be further manually limited by the operator. The constraints of mean heart dose and heart V_30_ should be set < 26 Gy and 45%. In our study, as θ_A_ decreases from 220° to 80°, the CI increased from 1.15 to 1.34 in HT. Our previous study also showed conformity became worse with more limitation of beam angle in HT^10^, which was similar with the present study. Therefore, further optimization would be needed to meet the constraints with limited θ_A_. Besides, we only simulated the dose distribution in esophageal cancer. The position of the esophagus is in the middle relative to other organs. The tumor of other organs needed to be verified further. More variables affecting lung volumes including organ motions and setup errors may exist in patients. The thorax anatomy of patients is not entirely symmetrical. The left and right lungs have different volumes. In Eq. (2) of our VBA, θ_RESL_ + θ_RESR_ = θ_RES_, could be fit for clinical application. The restricted angles on both sides of lungs could be unequal, however, the sum of the restricted angles on both sides (θ_RES_) would still be 360 − θ_A_ (Eq. 1). Our study was a preclinical study, which mainly used phantom images to establish the algorithm and verify the feasibility of the algorithm. Clinical retrospective cases study using this VBA algorithm is ongoing. Further clinical studies are needed to clarify these propositions in patients.

## Conclusion

This study successfully developed a VBA that can rapidly calculate the gantry arc angle to predict the lung V_5_. The operators can rapidly obtain the expected lung V_5_ with 20 iterations within 5 min. The developed algorithm can improve the efficiency of conventional radiotherapy planning.
